# Intrahepatic cholangiocarcinoma prognostic determination using pre-operative serum C-reactive protein levels

**DOI:** 10.1186/s12885-016-2827-7

**Published:** 2016-10-12

**Authors:** Zi-Ying Lin, Zhen-Xing Liang, Pei-Lin Zhuang, Jie-Wei Chen, Yun Cao, Li-Xu Yan, Jing-Ping Yun, Dan Xie, Mu-Yan Cai

**Affiliations:** 1 Sun Yat-sen University Cancer Center, State Key Laboratory of Oncology in South China; Collaborative Innovation Center for Cancer Medicine, Guangzhou, China; 2 Department of Pathology, Sun Yat-sen University Cancer Center, No. 651, Dongfeng Road East, 510060 Guangzhou, China; 3 Department of Prosthodontics, Sun Yat-Sen Memorial Hospital, Sun Yat-Sen University, Guangzhou, China; 4 Department of Pathology and Laboratory Medicine, Guangdong General Hospital, Guangzhou, China

**Keywords:** C-reactive protein, Intrahepatic cholangiocarcinoma, Prognosis

## Abstract

**Background:**

Serum C-reactive protein (CRP), an acute inflammatory response biomarker, has been recognized as an indicator of malignant disease progression. However, the prognostic significance of CRP levels collected before tumor removal in intrahepatic cholangiocarcinoma requires further investigation.

**Methods:**

We sampled the CRP levels in 140 patients with intrahepatic cholangiocarcinoma who underwent hepatectomies with regional lymphadenectomies between 2006 and 2013. A retrospective analysis of the clinicopathological data was performed. We focused on the impact of serum CRP on the patients’ cancer-specific survival and recurrence-free survival rates.

**Results:**

High levels of preoperative serum CRP were significantly associated with well-established clinicopathologic features, including gender, advanced tumor stage, and elevated carcinoembryonic antigen and carbohydrate antigen 19-9 levels (*P* < 0.05). Univariate analysis demonstrated a significant association between high levels of serum CRP and adverse cancer-specific survival (*P* = 0.001) and recurrence-free survival (*P* < 0.001). In patients with stage I/II intrahepatic cholangiocarcinoma, the serum CRP level was a prognostic indicator for cancer-specific survival. In patients with stage I/II or stage III/IV, the serum CRP level was a prognostic indicator for recurrence-free survival (*P* < 0.05). Additionally, multivariate analysis identified serum CRP level in intrahepatic cholangiocarcinoma as an independent prognostic factor (*P* < 0.05).

**Conclusions:**

We confirmed a significant association of elevated pre-operative CRP levels with poor clinical outcomes for the tested patients with intrahepatic cholangiocarcinoma. Our results indicate that the serum CRP level may represent a useful factor for patient stratification in intrahepatic cholangiocarcinoma management.

## Background

Cholangiocarcinoma is a relatively rare neoplasm acquired by humans. Recently, high incidence rates have been reported in Eastern Asia, and especially in Thailand [1]. Based on the location in the body where it develops, cholangiocarcinoma is further classified into intrahepatic, perihilar extrahepatic, or distal extrahepatic. Intrahepatic cholangiocarcinoma (IHCC) originates from the second segment of the bile duct, and is the least common of the cholangiocarcinoma classifications that a person could acquire. It accounts for 8–10 % of total cholangiocarcinoma cases diagnosed [2]. Its etiology is unknown, although various risk factors, including primary sclerosing cholangitis [3], liver fluke infestation [4], hepatolithiasis [5], and hepatitis viruses [6, 7], have been identified. These risk factors all induce a chronic inflammation in the biliary epithelium and partially obstruct the bile duct [8]. These risk factors are considered to be favorable for potential cancer development [8]. IHCC is seemingly incurable, has a rapid progression, and is lethal in most cases, with the 5-years survival rate being less than 5 % for non-resectable cases [9]. Surgical resection offers only a chance to cure IHCC, but the outcomes vary widely across affected patients.

Several prognostic factors have been identified for the prediction of IHCC patient survival. These factors include staging [10], para-aortic lymph node status [11], positive node to the total node ratio [12], tumor size, and the presence of multiple tumors [13]. Other novel molecular biomarkers, such as hepatoma-derived growth factor [14], SOX4 [15], loss of FBXW7 expression [16], Homer1 [17], and inactivation of Smad4 [18], seem to be associated with poor IHCC patient prognosis. Despite these critical association findings, the majority of these histological predictors only apply to assessments conducted after surgical intervention. Consequently, there is an urgent need to identify pre-treatment prognostic markers that can be used for an improved risk stratified treatment approach for patients that have not undergone surgical intervention.

Serum C-reactive protein (CRP), an acute phase reactive protein, has been defined as an inflammatory biomarker produced in response to pro-inflammatory cytokine hepatocyte stimulation [19]. As a result, CRP is closely associated with the development and outcome of many diseases [19]. During the past decade, elevated serum CRP has been associated with poorer prognosis in patients with various malignant cancers, including gastric cancer, colorectal cancer, breast cancer, and urological cancer [20–25]. This linkage implies a close association between inflammation and malignancy. A recent study has also revealed that chronic inflammation and pro-inflammatory cytokines, like interleukin 6 (IL-6), play an important role in the development and progression of cholangiocarcinoma [26]. Based on these conclusions, it seems as though CRP may be a promising prognostic factor that could be incorporated into prognostic models to improve the predictive accuracy of the outcomes for IHCC patients. Already, a retrospective study has shown that the serum CRP was a prognostic factor in a patient with a small size perihilar cholangiocarcinoma [27]. In published literature, the relation between serum CRP and prognosis of IHCC has not been explored yet. In this study, we aimed to explore the prognostic significance of pre-treatment serum CRP levels on cancer-specific survival (CSS) and recurrence-free survival (RFS) in IHCC patients.

## Methods

### Patients

This retrospective study included 140 IHCC patients that were treated at Sun Yat-sen University Cancer Center between the years of 2006 and 2013. The patient cases were selected for inclusion in this study based on the following criteria: pathological diagnosis of IHCC, primary and curative tumor resection surgery without preoperative anticancer treatment, the availability of preoperative serum CRP levels, liver function and clinicopathological and follow-up data. The IHCC cohort included 93 (66 %) men and 47 (34 %) women with a mean age of 54.11 years. The average follow-up time after surgery was 20.9 months (median: 14.7 months; range: 0.55 to 87.9 months). Follow-up evaluations were performed every 3 months within the first year, every 6 months for the next 2 years, and annually 3 years after the surgery. The histopathological findings of this IHCC cohort were also reviewed, including tumor multiplicity, intraepithelial ductal spread and vascular invasion. Tumor differentiation was determined based on the criteria proposed in the WHO Classification of Tumours of the Digestive System (2010 version). At the T stage, the lymph node status and the tumor stage were defined according to the UICC/AJCC tumor-node-metastasis (TNM) Classification System (2010 version). Ultrasonography, computed tomography (CT), or magnetic resonance imaging (MRI) scanning were used to detect tumor recurrence, which included incidences of intrahepatic recurrence or metastasis. The time of detection was used as the time of recurrence. In our study, the RFS was defined as the time from surgery to IHCC recurrence or patient death from IHCC, whichever came first in each individual case. The CSS was the time from the date of surgery to the last follow-up visit or date of death from IHCC. The Institute Research Medical Ethics Committee of Sun Yat-sen University Cancer Center approved the methods used in this study.

### Detection of serum CRP

Serum CRP levels were detected by the biosensor. To be included in the study, the serum used for CRP detection needed to be collected within 5 days before the tumor resection surgery. The biosensor detection system was adjusted regularly with a calibration curve acquired from the four-parameter Logit log mode. All the reagents used in CRP detection were derived from the WHO standards.

### Statistical analysis

A receiver operating characteristic (ROC) curve analysis was used to determine the preoperative serum CRP cutoff value that was acceptable for patient case inclusion in this study. The correlation between preoperative serum CRP level and the clinicopathologic features of the IHCC patients was evaluated by a *χ* ^2^ test. Using univariate analysis, survival curves were constructed using the Kaplan-Meier method. The differences between groups when considering survival were analyzed by the log-rank test. The effect of preoperative serum CRP and other clinicopathological variables on CSS and RFS were evaluated with the Cox proportional hazards regression model. For the variables with statistical significance in the univariate analysis, a further multivariate survival analysis was performed with the Cox regression model. The corresponding hazard ratio (HR) and 95 % confidence interval (CI) were extracted from the Cox regression models. All statistical analyses were performed using the SPSS statistical software package (SPSS Standard version 16.0, SPSS Inc.). A *P* < 0.05 in a two-sided analysis was considered to be statistically significant.

## Results

### Patient characteristics

All patients underwent curative resection for IHCC with the following intra-operative goals: complete tumor resection with lymphoadenectomy and leaving the cut surface free of tumor. Median tumor size was 5.5 cm (range from 0.5 to 15.0 cm). In this study, 140 IHCCs were located in right lobe (74, 53 %), left lobe (47, 34 %), both right and left lobes (17, 12 %), and quadrate lobe (2, 1 %), respectively. Most patients exhibited well-differentiated or moderately-differentiated tumors (*n* = 91, 65 %). Vascular invasion was observed in 54 (39 %) patients. Intraepithelial ductal spread was detected in 88 of 140 (63 %) patients with IHCC. The elevated levels of preoperative serum alanine aminotransferase (ALT) and aspartate aminotransferase (AST) were observed in 53 (38 %) and 43 (31 %) patients, respectively. Cancer recurrence was observed in 87 patients, including 55 of intrahepatic relapse, 17 of multiple metastases, 12 of lymph node metastasis and 3 lung metastases. Patients received postoperative chemotherapy according to the status of lymph node metastasis and multiple tumor nodules. The characteristics and pathological features of IHCC patients are detailed in Table 1.

**Table 1:** Correlation of preoperative serum C-reactive protein levels with patients’ clinico-pathological features in intra-hepatic cholangiocarcinoma

Variable	All cases	Serum CRP mg/L		*P* value^a^
		≤1.8	> 1.8	
Age (years)				0.355
≤ 55^b^	75	21 (28.0 %)	54 (72.0 %)	
> 55	71	21 (32.3 %)	44 (67.7 %)	
Gender				0.018
Male	93	22 (23.7 %)	71 (76.3 %)	
Female	47	20 (42.6 %)	27 (57.4 %)	
Location				0.263
Right lobe	75	26 (34.7 %)	49 (65.3 %)	
Left lobe	46	13 (28.3 %)	33 (71.7 %)	
Both lobes or quadrate lobe	19	3 (15.8 %)	16 (84.2 %)	
ALT				< 0.001
Normal	87	37 (42.5 %)	50 (57.5 %)	
Elevated	53	5 (9.4 %)	48 (90.6 %)	
AST				0.002
Normal	97	37 (38.1 %)	60 (61.9 %)	
Elevated	43	5 (11.6 %)	38 (88.4 %)	
HbsAg				0.417
Positive	55	15 (27.3 %)	40 (72.7 %)	
Negative	82	25 (30.5 %)	57 (69.5 %)	
CEA (ng/ml)				0.047
≤ 5	101	35 (34.7 %)	66 (65.3 %)	
> 5	38	7 (16.7 %)	31 (81.6 %)	
CA19-9 (U/ml)				0.024
≤ 35	57	23 (40.4 %)	34 (59.6 %)	
> 35	82	19 (23.2 %)	63 (76.8 %)	
Tumor size (cm)				0.119
≤ 5.5^c^	71	25 (35.2 %)	46 (64.8 %)	
> 5.5	69	17 (24.6 %)	52 (75.4 %)	
Nodal metastasis				0.158
Yes	29	6 (20.7 %)	23 (79.3 %)	
No	111	36 (32.4 %)	75 (67.6 %)	
Tumor multiplicity				0.830
Single	108	32 (29.6 %)	76 (70.4 %)	
Multiple	32	10 (31.2 %)	22 (68.8 %)	
Intraepithelial ductal spread				0.039
Absent	52	21 (40.4 %)	31 (59.6 %)	
Present	88	21 (23.9 %)	67 (76.1 %)	
Vascular invasion				0.940
Absent	86	26 (30.2 %)	60 (69.8 %)	
Present	54	16 (29.6 %)	38 (70.4 %)	
Grade				0.185
I	14	7 (50.0 %)	7 (50.0 %)	
II	77	23 (29.9 %)	31 (70.1 %)	
III	49	12 (24.5 %)	37 (75.5 %)	
TNM				0.011
I-II	78	30 (38.5 %)	48 (61.5 %)	
III- IV	62	12 (19.4 %)	50 (80.6 %)	
Postoperative chemotherapy				0.078
Yes	47	10 (21.3 %)	37 (78.7 %)	
No	93	32 (34.4 %)	61 (65.6 %)	

^a^Chi-square test

^b^Median age

^c^Median size

*CRP* C-reactive protein, *ALT* alanine aminotransferase, *AST* aspartate aminotransferase, *HbsAg* hepatitis B surface antigen, *CEA* carcinoembryonic antigen, *CA19-9* carbohydrate antigen 19-9, *TNM* tumor-node-metastasis

### Preoperative serum CRP level cutoff selection

**Fig. 1:**
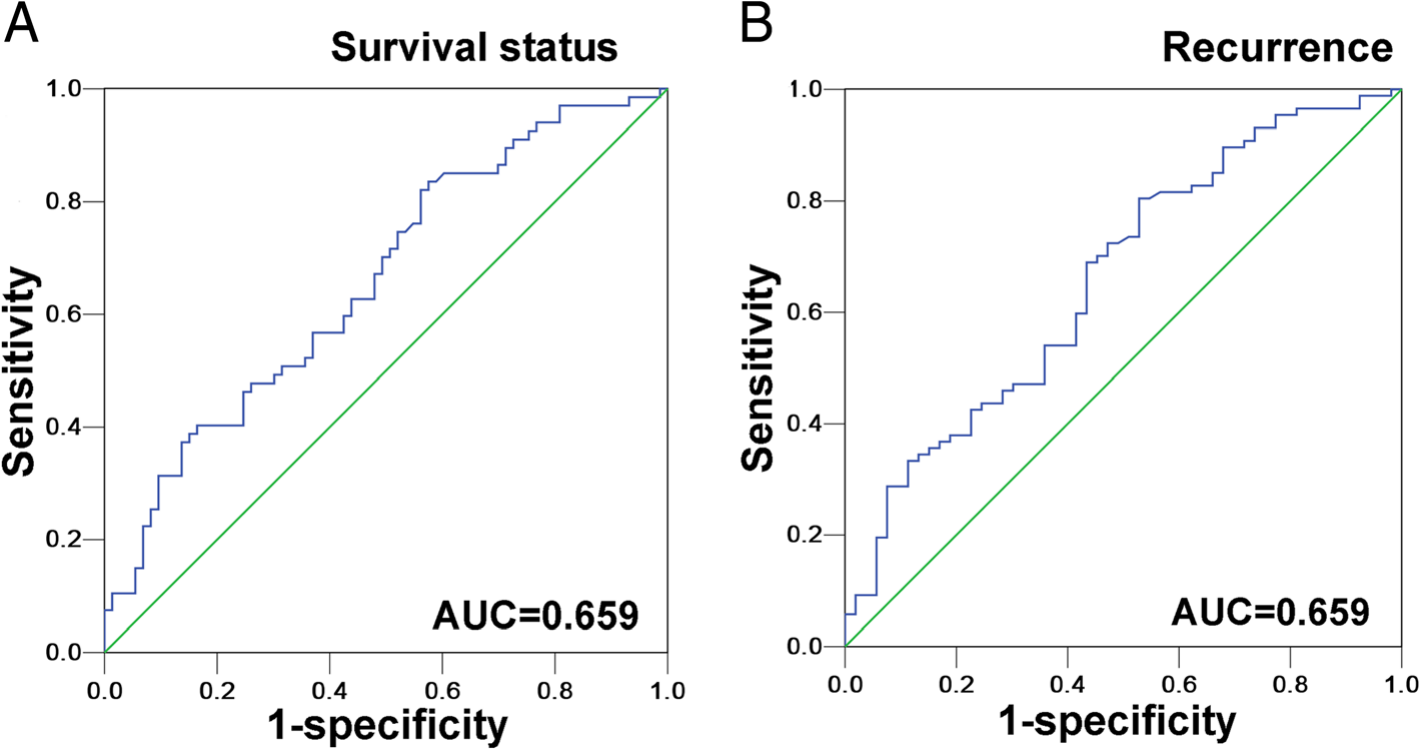
Receiver operating characteristic curve analysis determination of cutoff score for preoperative serum C-reactive protein levels. The sensitivity and specificity for each outcome were plotted: cancer-specific survival **a**, recurrence-free survival **b**

The mean pre-treatment plasma CRP level was 15.2 mg/L. To develop an optimal serum CRP cutoff value for further analysis, we subjected the serum CRP levels to an ROC curve analysis with respect to the survival and recurrence statuses (Fig. 1). The ROC curves showed the point on the curve closest to (0.0, 1.0), which maximizes both the sensitivity and specificity for the outcomes. Patients with cancer with CRP levels above the obtained cutoff value have a higher risk of tumor recurrence and cancer-related death than cases with levels below the value. Based on the gathered data, an optimal CRP level cutoff value of 1.8 mg/L was determined to differentiate between the opposing patient prognoses (area under the curve: 0.659; 95 % CI: 0.570–0.749) (Fig. 1a) as well as between the tumor recurrence and no further incidence of tumors (area under the curve: 0.659; 95 % CI: 0.566–0.752) (Fig. 1b).

### Relationship between preoperative serum CRP level and IHCC patient clinicopathological features

We separated patients into two groups according to low CRP levels (≤1.8 mg/L) or high CRP levels (>1.8 mg/L) according to the ROC curve analysis. We evaluated the associations between preoperative serum CRP levels and other clinicopathological factors gathered in the individual patient cases. An elevated serum CRP level was significantly correlated with gender, advanced tumor stage, elevated ALT, AST, carcinoembryonic antigen (CEA), carbohydrate antigen 19-9 (CA19-9) levels and intraepithelial ductal spread (*P* < 0.05). No significant associations were found in between CRP level and age, tumor location, hepatitis B virus infection, tumor size, tumor grading, nodal metastasis, tumor multiplicity, vascular invasion and chemotherapy administration (Table 1).

### Relationship between preoperative serum CRP level and IHCC patient survival

To investigate whether preoperative serum CRP level and other clinicopathological factors are associated with IHCC patient survival, we calculated univariate Cox proportional models for the CSS and RFS. Univariate analyses identified tumor size (≤5.5 *vs* >5.5 cm, *P* = 0.001), a high tumor stage (stage I/II *vs* III/IV, *P* = 0.007), nodal metastasis (absent *vs* presence, *P* = 0.011), vascular invasion (absent *vs* presence, *P* = 0.001), elevated CA19-9 (≤35 vs >35 U/ml, *P* = 0.016), and a high serum CRP level (≤1.8 vs >1.8 mg/L, *P* = 0.001) as poor prognostic factors for CSS. For a poor prognosis of RFS, having a larger tumor size (≤5.5 vs >5.5 cm, *P* = 0.001), a high tumor stage (stage I/II vs III/IV, *P* = 0.025), nodal metastasis (absent vs no presence, *P* = 0.016), vascular invasion (absent *vs* presence, *P* = 0.003), postoperative chemotherapy (chemotherapy vs on postoperative treatment, *P* < 0.001), and a high serum CRP level (≤1.8 vs >1.8 mg/L, *P* < 0.001) were identified as poor prognostic factors. Age, gender, tumor location, grading, elevated ALT, AST and CEA levels, and tumor multiplicity were not significantly associated with clinical outcomes for the set of patients (Table 2).

**Table 2:** Univariate analyses of serum C-reactive protein (CRP) levels and clinicopathologic variables in 140 patients with intrahepatic cholangiocarcinoma (Cox proportional-hazards regression)

Variables	All cases	Cancer-specific survival		Recurrence-free survival	
		Hazard Ratio (95 % CI)	*P* value	Hazard Ratio (95 % CI)	*P* value
Age (years)					
≤ 55^a^	75	1		1	
> 55	65	0.930 (0.572–1.513)	0.772	0.862 (0.562–1.321)	0.496
Gender					
Male	93	1		1	
Female	47	0.770 (0.455–1.301)	0.329	0.770 (0.488–1.214)	0.216
Location					
Right lobe	75	1		1	
Left lobe	46	1.329 (0.772–2.289)	0.304	1.224 (0.760–1.972)	0.407
Both lobes or quadrate lobe	19	1.192 (0.855–1.662)	0.300	1.217 (0.900–1.646)	0.202
ALT					
Normal	87	1		1	
Elevated	53	1.295 (0.794–2.110)	0.300	1.218 (0.791–1.874)	0.371
AST					
Normal	97	1		1	
Elevated	43	1.305 (0.783–2.175)	0.308	1.149 (0.729–1.812)	0.550
HbsAg					
Positive	55	1.027 (0.629–1.675)	0.916	1.212 (0.785–1.871)	0.385
Negative	82	1		1	
Tumor size (cm)					
≤ 5.5^b^	71	1		1	
> 5.5	69	2.315 (1.407–3.810)	0.001	2.072 (1.347–3.188)	0.001
Grade					
I	14	1		1	
II	77	0.490 (0.169–1.418)	0.188	0.363 (0.141–0.934)	0.036
III	49	0.910 (0.544–1.521)	0.719	0.794 (0.507–1.244)	0.314
Nodal metastasis					
No	111	1		1	
Yes	29	2.048 (1.169–3.589)	0. 011	1.847 (1.123–3.038)	0. 016
Tumor multiplicity					
Single	108	1		1	
Multiple	32	1.266 (0.729–2.198)	0.403	1.214 (0.742–1.985)	0.440
Intraepithelial ductal spread					
Absent	52	0.821 (0.507–1.329)	0.422	0.897 (0.585–1.375)	0.618
Present	88	1		1	
Vascular invasion					
Absent	86	1		1	
Present	54	2.298 (1.416–3.731)	0.001	1.901 (1.238–2.918)	0.003
TNM					
I-II	78	1		1	
III-IV	62	1.977 (1.203–3.249)	0.007	1.644 (1.065–2.536)	0.025
CEA (ng/ml)					
≤ 5	101	1		1	
> 5	38	1.276 (0.734–2.219)	0.388	1.317 (0.819–2.119)	0.255
CA19-9 (U/ml)					
≤ 35	57	1		1	
> 35	82	1.889 (1.125–3.169)	0.016	1.441 (0.930–2.234)	0.102
Postoperative chemotherapy					
No	93	1		1	
Yes	47	1.520 (0.925–2.498)	0.098	2.515 (1.619–3.908)	<0.001
Serum CRP (mg/L)					
≤ 1.8	42	1		1	
> 1.8	98	2.965 (1.551–5.667)	0.001	2.751	<0.001

*ALT* alanine aminotransferase, *AST* aspartate aminotransferase, *HbsAg* hepatitis B surface antigen, *CEA* carcinoembryonic antigen, *CA19-9* carbohydrate antigen 19-9, *TNM* tumor-node-metastasis

^a^Median age

^b^Median size

**Fig. 2:**
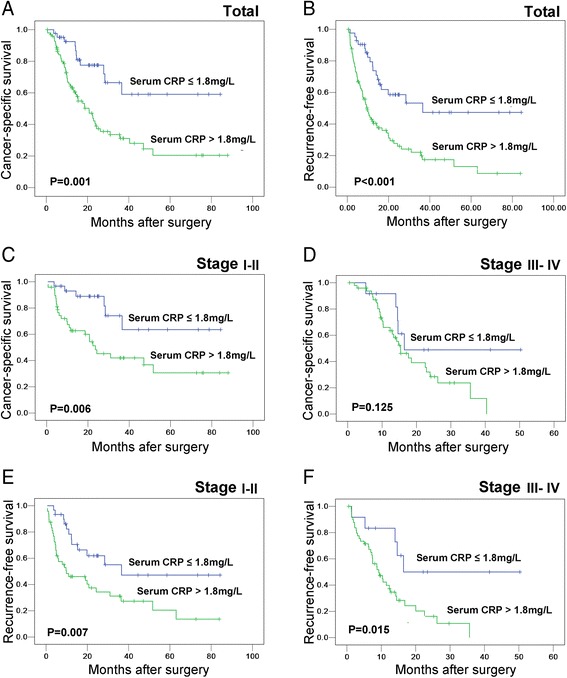
Kaplan-Meier survival analysis of preoperative serum C-reactive protein (CRP) levels in patients with intrahepatic cholangiocarcinoma (IHCC) (log-rank test). The preoperative serum CRP levels and the probability of cancer-specific survival (CSS, **a**) and recurrence-free survival (RFS) of IHCC patients **b** The preoperative serum CRP levels and the probability of cancer-specific survival in IHCC patients at stage I-II **c** and stage III-IV **d** The preoperative serum CRP levels and the probability of RFS in IHCC patients at stage I-II **e** and stage III-IV **f**

Among the 140 IHCC patients, death occurred in 11 of 42 (26 %) patients with a low serum CRP level and in 56 of 98 (57 %) patients with a high serum CRP level (*P* = 0.001). In the Kaplan–Meier survival analysis, there was highly significant association between a high serum CRP level and shortened patient survival time (*P* = 0.001, Kaplan-Meier Method) (Fig. 2a). A stratified survival analysis was also performed to evaluate the serum CRP levels in subsets of the IHCC patients that were at different clinical stages. Our results demonstrated that a high serum CRP level was a prognostic factor in IHCC patients with stage I/II cancer (*P* = 0.006, Kaplan-Meier Method) (Fig. 2c) but not stage III/IV cancer (*P* = 0.126, Kaplan-Meier Method) (Fig. 2d).

Results in the RFS analysis were similar to that in CSS analysis. Patients with high serum CRP level showed a significant trend toward worse RFS compared to the RFS of patients with low serum CRP levels (*P* < 0.001, Kaplan-Meier Method) (Fig. 2b). Additionally, a stratified survival analysis showed that a high serum CRP level was a predictor for RFS in both stage I/II cancers (*P* = 0.007, Kaplan-Meier Method) (Fig. 2e) and stage III/IV cancers (*P* = 0. 015, Kaplan-Meier Method) (Fig. 2f).

### Independent prognostic value of preoperative serum CRP levels in IHCC patients

To determine the independent prognostic value of the serum CRP levels for CSS and RFS, we performed multivariate analyses using a Cox proportional hazard model. The clinicopathologic variables, specifically tumor stage, tumor size, nodal metastasis, vascular invasion, CA19-9 level, administration of postoperative chemotherapy, and serum CRP levels, were tested in the multivariate analyses. The clinicopathological variables were found to be of statistical significance in the univariate analyses. In the multivariate analyses, we found that high serum CRP level was a prognostic factor for poor CSS (*P* = 0.004) and RFS (*P* < 0.001) of IHCC patients. Additionally, it appeared that independent of tumor stage, nodal metastasis and CA19-9 level were not prognostic factors for poor CSS and RFS (Table 3). Despite this finding, tumor size and vascular invasion were found to be independent prognostic predictors for poor CSS and RFS, as well as administration of postoperative chemotherapy for poor RFS (*P* < 0.05) (Table 3).

**Table 3:** Cox multivariate analyses of prognostic factors on patients’ survival

Variables	Hazards ratio	95 % CI	*P* value
Cancer-specific survival^a^			
CA19-9, U/ml (≤35 *v* >35)	1.553	0.904–2.668	0.111
Tumor size, cm (≤5.5 *v* >5.5)	2.143	1.239–3.706	0.006
Nodal metastasis (absent *v* present)	1.390	0.703–2.749	0.343
Vascular invasion (absent *v* present)	1.954	1.181–3.233	0.009
TNM (I*-*II *v* III*-*IV)	0.884	0.459–1.702	0.711
Serum CRP, mg/L (≤1.8 *v* >1.8)	2.646	1.356–5.164	0.004
Recurrence- free survival^b^			
Postoperative chemotherapy (yes *v* no)	1.788	1.042–3.067	0.035
Tumor size, cm (≤5.5 *v* >5.5)	1.920	1.153–3.197	0.012
Nodal metastasis (absent *v* present)	1.277	0.689–2.367	0.437
Vascular invasion (absent *v* present)	1.733	1.107–2.715	0.016
TNM (I*-*II *v* III*-*IV)	0.663	0.369–1.191	0.169
Serum CRP, mg/L (≤1.8 *v* >1.8)	2.827	1.636–4.886	< 0.001

*CI* confidence interval, *CA19-9* carbohydrate antigen 19-9, *TNM* tumor-node-metastasis, *CRP* C-reactive protein

^a^The total number of patients and total number of events in this model were 140 and 67, respectively

^b^The total number of patients and total number of events in this model were 140 and 87, respectively

## Discussion

CRP is an acute-phase reactant, and plays a role in microbial infection, trauma, infarction, autoimmune diseases, and malignant cancers [28]. Recently, high levels of serum CRP have been associated with metastatic disease and a poor prognosis in various malignant cancers, including perihilar cholangiocarcinoma. However, the role of CRP status in IHCC, and its utility to clinicians, remains unknown. In our study, we retrospectively assessed IHCC patient cases in order to explore the prognostic value of preoperative CRP. We sought to determine the relative survival rates for IHCC patients who underwent curative operations to remove cancerous tumors.

Our study demonstrated that preoperative serum CRP levels were strongly associated with adverse CSS and RFS in IHCC patients. Furthermore, the preoperative serum CRP level appeared independent from tumor characteristics and treatment allocation. Other groups have reported similar results [23, 29–32], in which preoperative serum CRP level was significantly correlated with other pathologic parameters found in solid cancers, such as decreased survival times, increased loco-regional recurrence rates, more severe post-operative complications, and a shorter relapse time after surgery. A more recent study has found that C-reactive protein, at the time of diagnosis, predicts poorer outcomes in hepatocellular carcinoma patients that are unable to have tumor removal surgeries [33]. More importantly, high CRP level was significantly correlated with poor survival and found to be independently predictive of survival in patients with perihilar cholangiocarcinoma as evidenced by the in the univariate and multivariate analyses [27]. In summation, it appears the pre-operative CRP level may be useful prognostic predictor in many cancer patients who have undergone a radical treatment to remove the cancer. Despite this finding, the prognostic significance in IHCC patients that have not undergone tumor removal surgery still needs further study.

In our study, subgroup analyses, with respect to the TNM stage, supported the prognostic relevance of serum CRP, except for the CSS rate in the subgroup of TNM stage III–IV. This finding is inconsistent with the previous studies, which imply a strengthened association between serum CRP concentration and prognosis in patients with advanced cancers, included colorectal cancer, gastric cancer, ovarian cancer, and renal cell cancer [20, 34–37]. Notably, there is also a report [38] which shows no significant prognostic value when considering serum CRP level for predicting cancer recurrence rate in stage II and III colorectal cancer. Taken together, differences in the geographic backgrounds, biological characteristics of different tumors, selection of the CRP cutoff valve, patient heterogeneity, small sample sizes, and different definitions of end points (disease-free, cancer specific survival, or overall survival) may contribute to these discrepancies.

The underling mechanism regarding the elevated CRP level indicating a potential poorer outcome for cancer patients is unknown. There are three possible explanations. First, this phenomenon could be explained by causality, or that an elevated CRP level promotes tumor progression. Second, this could be explained by reverse causality, or that tumor progression increases the CRP level. Lastly, there could be a confounding explanation. A third factor, for example inflammation, could increase both CRP level and tumor malignancy. Notably, there are evidence [26] that the inflammatory field effect, reflected by elevated CRP, may be directly involved in tumor progression. This could explain the CRP level’s prognostic significance in IHCC, which has a development and progression that are closely related with inflammation [39, 40]. For example, IL-6, one of the main inducers of CRP production, has been shown to be associated with development and progression of cholangiocarcinoma and other cancers [28, 41]. Moreover, CRP was shown to directly enhance tumor cell proliferation under stressed conditions in a recent study on myeloma [42]. Clearly, the causal mechanisms of CRP regarding the progression of IHCC and other cancers need to be clarified in further investigations.

The findings presented in this report may have impacts for the design of future clinical trials. Most studies in advanced IHCC only stratify prognoses according to variables like tumor staging and grading, presence or absence of vascular invasion/extra-hepatic spread, and CEA or CA19-9 levels. The strong and independent prognostic significance of serum CRP levels found in this study provides evidence to collect serum CRP levels in future clinical trials for the potential determination of an IHCC patient’s prognosis.

There are a few limitations in the design of this study. CRP is known to be a non-specific marker of inflammation. It is possible that an undocumented super infection could influence CRP concentrations in the serum. Furthermore, as IHCC is mainly located within the liver, the synthetic function of the hepatocytes may be affected. This change in functioning may act as a confounding factor that correlates directly with both the development of IHCC and the serum concentration of CRP. Moreover, our study is limited by its retrospective nature and a rather heterogeneous group of patients.

Based on the results of our study, it is possible that a higher preoperative CRP level in IHCC patients was associated with a higher risk for recurrence and earlier death because of the disease. Whether patients can be selected for resection, or increase the chances of curative resection, can only be evaluated in a controlled prospective clinical trial. However, to the best of our knowledge, our study represents the first one validates the prognostic value of serum CRP levels in IHCC patient prognoses.

## Conclusions

In conclusion, the preoperative CRP level was a strong and independent predictor of a poor prognostic outcome, as indicated by the univariate and multivariate analyses. Adding the serum CRP into TNM stage factor could improve the ability to discriminate between IHCC patients’ outcomes. Our data seem to indicate that CRP could function as an independent prognostic factor of outcomes in IHCC. Additionally, the data support the consideration of the preoperative CRP level for therapy stratification. The causal role of CRP in tumor progression merits further investigation in preclinical studies.

## Abbreviations

CRP: C-reactive protein
CSS: Cancer-specific survival
IHCC: Intrahepatic cholangiocarcinoma
RFS: Recurrence-free survival
ROC: Receiver operating characteristic
